# Optimum Design of PI^*λ*^D^*μ*^ Controller for an Automatic Voltage Regulator System Using Combinatorial Test Design

**DOI:** 10.1371/journal.pone.0166150

**Published:** 2016-11-09

**Authors:** Bestoun S. Ahmed, Mouayad A. Sahib, Luca M. Gambardella, Wasif Afzal, Kamal Z. Zamli

**Affiliations:** 1 Istituto Dalle Molle di Studi sull’Intelligenza Artificiale (IDSIA),CH-6928 Manno-Lugano, Switzerland; 2 Software and Informatics Engineering Department, Engineering College, Salahaddin University - Erbil, Kurdistan Region, Iraq; 3 School of Innovation, Design and Engineering, Mälardalen University, Västerås, Sweden; 4 IBM Centre of Excellence, Faculty of Computer Systems and Software Engineering, Universiti Malaysia Pahang Lebuhraya Tun Razak, 26300 Kuantan, Pahang Darul Makmur, Malaysia; Nankai University, CHINA

## Abstract

Combinatorial test design is a plan of test that aims to reduce the amount of test cases systematically by choosing a subset of the test cases based on the combination of input variables. The subset covers all possible combinations of a given strength and hence tries to match the effectiveness of the exhaustive set. This mechanism of reduction has been used successfully in software testing research with *t*-way testing (where *t* indicates the interaction strength of combinations). Potentially, other systems may exhibit many similarities with this approach. Hence, it could form an emerging application in different areas of research due to its usefulness. To this end, more recently it has been applied in a few research areas successfully. In this paper, we explore the applicability of combinatorial test design technique for Fractional Order (FO), Proportional-Integral-Derivative (PID) parameter design controller, named as FOPID, for an automatic voltage regulator (AVR) system. Throughout the paper, we justify this new application theoretically and practically through simulations. In addition, we report on first experiments indicating its practical use in this field. We design different algorithms and adapted other strategies to cover all the combinations with an optimum and effective test set. Our findings indicate that combinatorial test design can find the combinations that lead to optimum design. Besides this, we also found that by increasing the strength of combination, we can approach to the optimum design in a way that with only 4-way combinatorial set, we can get the effectiveness of an exhaustive test set. This significantly reduced the number of tests needed and thus leads to an approach that optimizes design of parameters quickly.

## Introduction

Combinatorial test design techniques can significantly reduce the number of test cases. They are an alternative method to exhaustive testing by allowing a minimized set of tests to represent the actual set of test cases based on *t*-way covering criteria (where *t* represents the desired interaction strength of combinations). For example, an exhaustive test set for a system with 10 Boolean input parameters needs 1024 cases whereas it needs only 13 cases with 2-way set.

Combinatorial test design brings mainly two benefits for the system-under-test. First, it will reduce the amount of test cases dramatically, which in turn reduces the time taken for testing. Second, the combinatorial set will examine how the system reacts under different circumstances and scenarios. Owing to these benefits, recently, this technique has been applied to several different domains. For example, Cohen et al. [1] apply this technique successfully to test configurable software systems in the presence of constraints. Wang et al. [2] use this technique to build navigation graphs for dynamic web applications. Wang et al. [3] also used it for security systems to detect buffer overflow vulnerabilities. Borodai and Grunskii [4] used it for hardware testing and Lei et al. [5] applied it for concurrency testing. Sahib et al. [6] apply the pairwise method to control DC servo motors. Shasha et al. [7] used it for gene expression regulation and Hoskins et al. [8] used it for performance evaluation of communication systems.

Given the aforementioned benefits, in this paper, we adopt the combinatorial test design technique in the application domain of control systems to design an optimum Fractional Order Proportioanl-Integral-Derivative controller (FOPID). The FOPID is tuned to improve the performance of an automatic voltage regulator (AVR) in power generation systems. The AVR is utilized to maintain the terminal voltage of a synchronous generator at a specified level.

FOPID is a generalized structure of the classical PID controller that uses the concept of fractional calculus, where the orders of the derivative and integral parts are non-integer values. A FOPID is identified by five parameters: a proportional gain, integral gain, derivative gain, integral order, and derivative order. Previous research results in various applications have shown that FOPID controller has an improved performance and robustness compared to conventional PID [9].

In the literature, many design methods have been reported to find the optimum FOPID parameters. These methods can be classified mainly into two types, analytical and heuristic optimization methods. Analytical based methods include; Pole distribution [10], frequency domain approach [11], state-space design [12], two-stage or hybrid approach [13] and piecewise orthogonal functions approach [14]. On the other hand, heuristic methods include; particle swarm optimization (PSO) [15], chaotic ant swarm (CAS) [9] and Genetic algorithm [16].

Commonly, each optimization algorithm is associated with a predefined bounded searching space spanned by the vectors of solution variables [17]. However, in this space, infinite number of feasible solutions exist [18]. As a result, infinite search spaces impairs the effectiveness and efficiency of the algorithm. In this paper, the combinatorial test technique is used to assist and improve the search algorithm in optimizing the FOPID parameters. The improvement lies in the reduction of the search space domain.

Given such a prospect, this paper proposes a new application of combinatorial test design. Our contributions can be summarized as follows:

- A new strategy based on combinatorial test design applied for FOPID parameter tuning.
- The research reports the first experimental results for combinatorial test design that indicates its practical use in this field.
- Different algorithms were especially designed to effectively generate and apply combinatorial tests for FOPID.
- Experimental results indicate that combinatorial test design can find the combinations that leading to optimum design.

### Combinatorial Test Design Concepts

**Fig 1:**
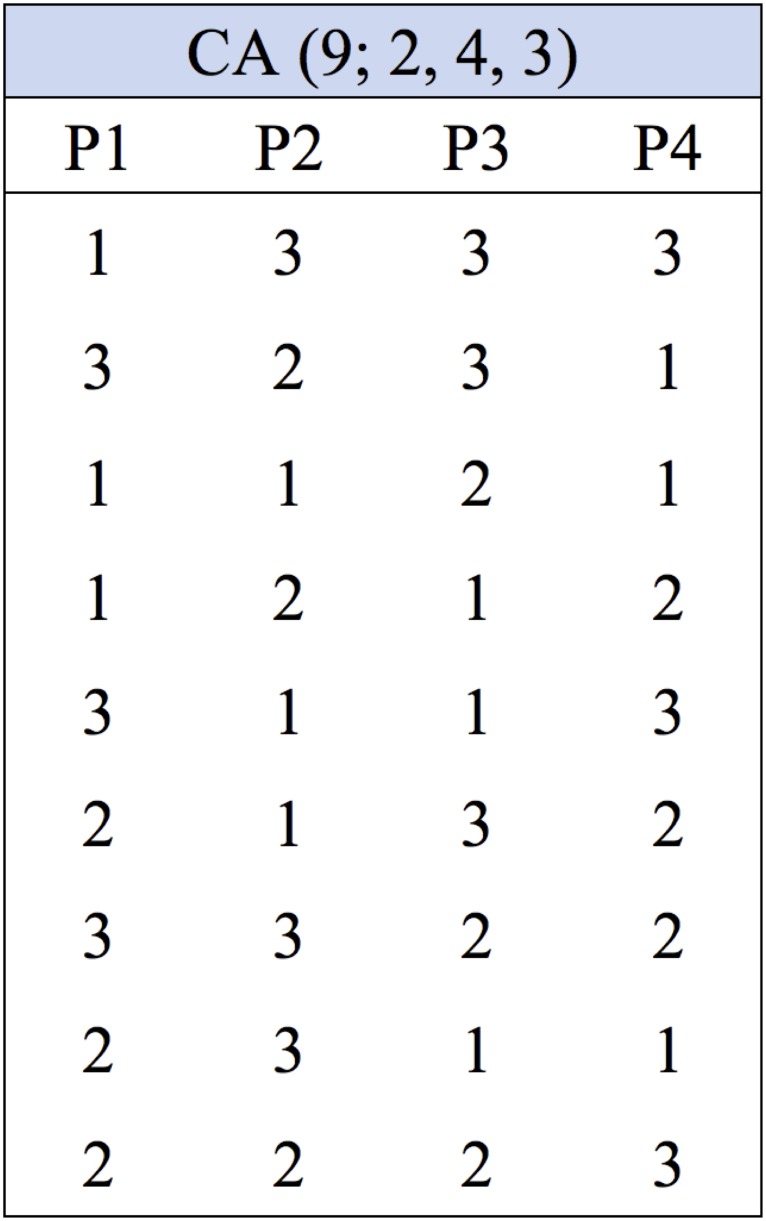
A Test Set Represented by CA Object.

Combinatorial test design is used as a sampling technique derived from a mathematical object called covering array (CA) [19]. CA can be illustrated as an array that contains all possible test cases. Each row in this array represents a test case, and each column represents an input-parameter. In general, CA can be defined as *CA* (*N*; *t*, *k*, *v*) where *N* represents the array size, *t* is the interaction strength of combinations, *k* is number of input-factors, and *v* is the the number of values for each input-factor [19]. Here the array can be seen as a test set of *N* × *k* array with *v* values for each *k* parameter where (*v* = 0, 1, 2, …, *v* − 1) in a way that every *N* × *t* sub-array (i.e., *t* − *tuples*) contains all ordered subsets from *v* of size *N* at least one times. Fig 1 shows an example of a test set represented by *CA* notation as *CA* (9; 2, 4, 3).

**Fig 2:**
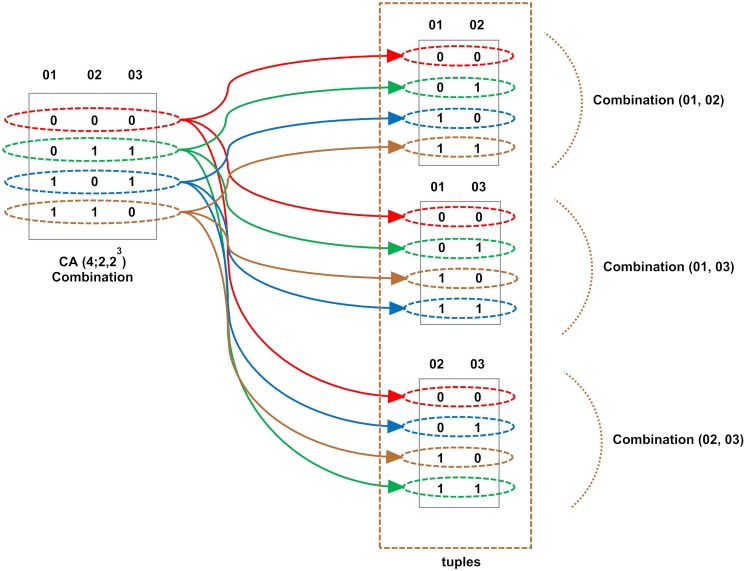
An Illustration for the Coverage Mechanism in Combinatorial Test Design.

Clearly, the test set in Fig 1 has a size of nine tests. The set is designed for a system with four input parameters, each of them having three values and the set takes the combination of two parameters. To use the exhaustive test set, there is a need for 3 × 3 × 3 × 3 = 81 test cases, whereas following the combinatorial test design method, there is a great reduction of the test cases number to only nine while all *t*-tuples has been covered by the set. To show the reduction and *t*-tuples coverage in the combinatorial test design method, we consider a simple example in Fig 2.

The test set in Fig 2 is a set for three input parameters in which each of them has two values (0 and 1). The test set can fulfill full coverage of the 2 − *tuples* by only four cases (i.e., *N* = 4). Combination among the input factors equals 12, [(A, B), (A, C), and (B, C) the result for each = 2^2^ = 4]. The left hand side array in the figure shows this test set. The first row in this set covers three tuples (i.e., red color tuples 25% of the 2 − *tuples*), thus only nine tuples remain in the tuples list that are shown on the right side of Fig 2. The next row covers three more tuples with green color tuples and totally with the previous row, 50% of the total tuples. This will continue until we reach 100% coverage of tuples, as shown in Fig 2. There could be constraints between these input parameters, however in this research there is no constraints between the tuned parameters.

This process becomes an NP-hard problem when the number of input parameters and their values grow. Hence, there is a need to design and implement efficient algorithms to generate the test set. In the coming sections, we will show how to generate this combinatorial set by showing different algorithms. In addition, we will discuss the input parameters, their type and values that we use for the case of FOPID controller tuning.

### Related Work

As mentioned previously, combinatorial test design techniques used to detect failures by testing interactions of input parameters through generation of a covering array (CA) test suite. The basic goal of such techniques is to cover every *t* − *tuple* of any input interaction of system under test at least once [1]. Two survey papers have been written on the topic of combinatorial testing strategies [20, 21] while Kuliamin and Petukhov [22] present a survey on the methods of constructing CAs. The methods for constructing CAs can be categorized into three categories [1, 19]: (1) algebraic methods (2) meta-heuristic methods and (3) greedy search methods. Algebraic methods use extremely fast mathematical techniques (both direct and recursive) [1] but their applicability is limited to certain special combinatorial test structures [19]. Examples of using algebraic methods for CA construction include those cited in [23, 24]. Meta-heuristic methods apply complex and iterative heuristic methods that include simulated annealing, tabu search, genetic algorithms, particle swarm and others. Although being computationally intensive, meta-heuristic methods have produced some CAs of the smallest size known. Examples of using meta-heuristic methods includes those cited in [19, 25, 26]. Greedy search methods are known to be faster than meta-heuristic search and are applicable to arbitrary test structures but may or may not produce smallest-size CAs. Examples of using greedy methods include those cited in [1, 27]. The three methods of CA generation are sometimes used in combination also. Examples of such integrated approaches include those cited in [28, 29].

The use of metaheuristic search techniques for CA generation is more recent where different optimization approaches have been proposed [26, 30, 31]. These optimization approaches typically start with a preexisting test set and then a series of transformations are applied to the test set until desirable combinations are covered. Besides simulated annealing, hill climbing, great flood, tabu search, particle swarm optimization, ant colony optimization and genetic algorithm, other search mechanisms are applicable for combinatorial optimization such as using evolutionary game dynamics [17, 18, 32].

Combinatorial test design has been used in various applications such as in optimal route planning for airlines, task scheduling, task allocation, network planning, gene expression regulation, performance evaluation of communication systems and hardware testing. In control system applications, tuning the parameters of a controller, such as the PID controller, has been proposed to represent another application of combinatorial optimization techniques [6]. In determining the optimal PID parameters, several heuristic methods have been introduced, such as genetic algorithm [16, 33], neural network [34], fuzzy based approach [35], particle swarm optimization [6, 15, 36] and chaotic ant swarm [9] techniques. Most systems arising in practice have time varying parameters which will affect the performance of the designed controller. In such cases a supervisory system can be used such that, when the performance of the controller drops below a prescribed level of acceptable performance index, the proposed optimization procedure must be reengaged. Therefore, the tuning process has to be sufficiently satisfactory in terms of convergence speed and this can be achieved when reducing the optimization searching space. In this paper, the combinatorial test technique is used to assist and improve the search algorithm in optimizing the FOPID parameters. The improvement lies in the reduction of the search space domain.

The performance of the PID controller can be enhanced by using the concept of fractional calculus in which the orders of the derivatives and integrals are non-integer. Based on this concept, the standard PID is generalized to FOPID. Designing an optimal FOPID involves the tuning of five parameters. Compared to PID, the tuning of FOPID is complicated and remains a challenging problem.

Recently, many optimization algorithms have been reported for the design of optimal FOPID. Such algorithms include genetic algorithm (GA) [16, 37], particle swarm optimization (PSO) [38], improved electromagnetism-like algorithm with genetic algorithm (IEMGA) [39], chaotic ant swarm (CAS) [9], artificial bee colony (ABC) [40], multi-objective extremal optimization (MOEO) [41], gases Brownian Motion Optimization (GBMO) [42], bacterial foraging optimization algorithm (BFOA) [43], and ant colony optimization (ACO) [37].

In all the aforementioned algorithms, the optimization search is performed within an open five dimensional space of parameters set. For each parameter, a real range of values is defined such as the ranges selected in this paper. The open space search delays the optimization process, consumes its effort, and may lead to local minimum problems. Therefore, we propose the combinatorial interaction design to reduce the space of search and to achieve an effective optimization process.

### System Model

**Fig 3:**
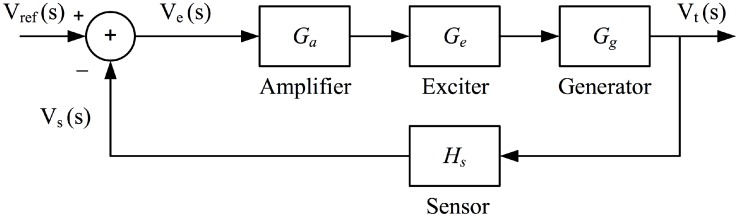
AVR system block diagram.

In a power system, disturbance such as sudden change in loads cause an oscillatory behavior around a set point terminal voltage of the synchronous generator. Such an oscillation affects the stability of the power system and degrades the efficiency of power transmission. To improve the dynamic stability of a power system and to increase its efficiency, the excitation systems of the synchronous generators are controlled using an automatic voltage regulator (AVR) system. The AVR system attempts to hold the terminal voltage of the synchronous generator at a specified level. A typical AVR system consists of four main components, namely amplifier (*G*_*a*_), exciter (*G*_*e*_), generator (*G*_*g*_), and sensor (*H*_*s*_). Each component is modeled by a first order system defined by a gain and a time constant. The arrangement of the AVR system components is shown in Fig 3

The terminal voltage Δ*V*_*t*_(*s*) of the generator is continuously sensed by the sensor and compared with the desired reference voltage Δ*V*_*ref*_(*s*). The difference between the reference and the sensed terminal voltages (error voltage Δ*V*_*e*_(*s*)) is amplified through the amplifier and used to excite the generator using the exciter. The transfer functions of *G*_*a*_, *G*_*e*_, *G*_*g*_, and *H*_*s*_ are:

$$G_{a}=\frac{K_{a}}{T_{a}s+1}$$ (1)

$$G_{e}=\frac{K_{e}}{T_{e}s+1}$$ (2)

$$G_{g}=\frac{K_{g}}{T_{g}s+1}$$ (3)

$$H_{s}=\frac{K_{s}}{T_{s}s+1}$$ (4)

The AVR system parameters considered in this work are; *K*_*a*_ = 10.0, *T*_*a*_ = 0.1, *K*_*e*_ = 1.0, *T*_*e*_ = 0.4, *K*_*g*_ = 1.0, *T*_*g*_ = 1.0, *K*_*s*_ = 1.0, *T*_*s*_ = 0.01 [9, 40, 44–46]. With these parameter values the closed loop transfer function of the AVR system becomes:

$$G_{A}VR=\frac{\Delta V_{t}\left(s\right)}{\Delta V_{r}ef\left(s\right)}=\frac{0.1s+10}{0.0004s^{4}+0.045s^{3}+0.555s^{2}+1.51s+11}$$ (5)

**Fig 4:**
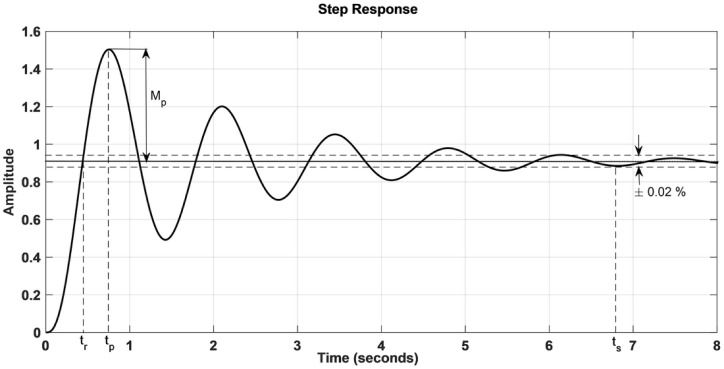
Step Response of the AVR system without controller.

The unit step response of the AVR system is shown in Fig 4.

From Fig 4 the AVR system possess an underdamped response with steady state amplitude value of 0.909, peak amplitude of 1.5 (*M*_*p*_ = 65.43%) at peak time *t*_*p*_ = 0.75, rise time *t*_*r*_ = 0.42 sec., settling time *t*_*s*_ = 6.97 sec. at which the response has settled to 98% of the steady state value.

The response of the AVR can be improved by utilizing a controller in the forward path. Commonly, a PID controller is employed for this task due to its simple structure. The performance of the PID controller can be enhanced by using the concept of fractional calculus, where the orders of derivatives and integrals are non-integer. The following section introduces the FOPID controller, along with its parameters and relevant equations.

### Fractional Order PID Controller

The idea of using a fractional-order controller for a dynamic system belongs to Oustaloup [47] who developed the so-called CRONE controller (CRONE is a French abbreviation of Non Integer Order Robust Control). Then, Podlubny [48] proposed a generalization of PID controller, which is called PI^λ^D^*μ*^ controller. The transfer function of the PI^λ^D^*μ*^ controller is given by

$$C_{PID}=K_{p}+\frac{K_{i}}{s^{\lambda }}+K_{d}s^{\mu }\;\;\left(\lambda ,\mu ,>0\right)$$ (6)

In practice, the fractional order Laplace operators (*s*^λ^ and *s*^*μ*^) in Eq (6) are approximated numerically with integer order transfer functions. The idea is to obtain an integer-order transfer functions whose behavior approximates the fractional orders. Oustaloup’s approximation is one of the available frequency-domain methods. It uses a recursive distribution of N poles and N zeros [47] defined by

$$s^{\alpha }\approx k\prod_{n=1}^{N}\frac{1+\frac{s}{w_{z,n}}}{1+\frac{s}{w_{p,n}}}\;\;\;0<\alpha <1$$ (7)

The approximation is valid within a predefined bandwidth defined by the frequency range [*ω*_*l*_, *ω*_*h*_]. The gain k is adjusted until both sides of Eq (7) have 0dB gain at *ω* = 1 rad/s. The approximation accuracy depends on the chosen number of poles and zeros (N). The approximation can be improved by increasing (N), however, this will be at the expense of computational complexity. The frequencies of the poles and zeroes in Eq (7) can be calculated recursively by

$$\varepsilon ={\left(\omega_{h}/\omega_{l}\right)}^{\alpha /N}$$ (8)

$$\eta ={\left(\omega_{h}/\omega_{l}\right)}^{(1-\alpha )/N}$$ (9)

$$\omega_{z,1}=\omega_{l}\sqrt{\eta }$$ (10)

$$\omega_{p,n}=\omega_{z,n}\varepsilon ,\;\;n=1,2,...,N$$ (11)

$$\omega_{z,n+1}=\omega_{p,n}\eta ,\;\;n=1,2,...,N-1$$ (12)

In case *α* > 1, the fractional order can be treated as

$$s^{\alpha }=s^{\left\lfloor \alpha \right\rfloor }\times s^{\alpha -\lfloor \alpha \rfloor }$$ (13)

where ⌊.⌋ denote the floor function. Thus, the fractional part (*α* − ⌊*α*⌋) can be approximated using Eq 7.

## Methods

### Test Design Procedure

In this section, we present the combinatorial approach to reach the optimum design of the controller. To generate this set, two levels of algorithms were needed. First, an algorithm to generate the combination of input parameters. Second, an algorithm to optimize the final set using the actual values’ set of the parameters. The following subsections illustrates each step of this procedure in detail.

### Input-Parameter Combination

This step represents the first step of the test design. The number of input-parameters of the system-under-test is determined first. Then, an algorithm will use these parameters to generate all combinations based on the combination strength provided. For low number of parameters, the combinations could be easily generated. However, when the number of parameters grows, the time for generation will grows exponentially. To avoid this situation, we have carefully designed an algorithm; Algorithm 1 shows the steps in detail.

**Algorithm 1**: Parameter Combination Generator

 **Input**: Input-parameters *k* and combination strength *t*

 **Output**: All *t*-combinations of *k* where *k* = *k*_1_, *k*_2_, *k*_3_, …, *k*_*n*_

1 Let Comb be an array of length *t*;

2 Let *i* be the index of Comb array;

3 Create a stack *S*;

4 *S* ← 0;

5 **while** *S* ≠ *null* **do**

6  *i* = (the length of *S* − 1);

7  *v* = pop the stack value;

8  **while** *pop value* < *k* **do**

9   set Comb of index (*i*) to *v*;

10   *i* ← *i* + 1;

11   *v* ← *v* + 1;

12   push *v* to stack;

13   **if** *i* = *t* **then**

14    Add Comb to final array;

15    break;

16   **end**

17  **end**

18 **end**

As shown in Algorithm 1, the algorithm takes *k* input parameters and produces t-combination (t-tuples) of them, each time adding the combinations to a final array containing all *t*-combinations of k. To avoid the enumeration of all n-bits, a stack data structure was used to hold the parameters permanently by “pushing” them into the stack and then “popping” them when needed during the iterations. Additionally, a temporary array was created with index i to help the generated combinations in each iteration (Steps 1–2). A stack data structure (*S*) was created and the first parameter (0) was pushed inside (Steps 3–4). The algorithm continues to iterate until the stack becomes empty (Step 5). The index number i of the Comb array was set to length of *S* − 1 and the value *v* of this index *i* was set to the top value in the stack (i.e. *pop*) until *v* was less than *k* (Steps 6–9). Furthermore, the algorithm continues to increment *i* and *v*, then puts the value of *v* into S until the index number equals to the length of the required interaction strength *t* (Steps 9–15).

**Fig 5:**
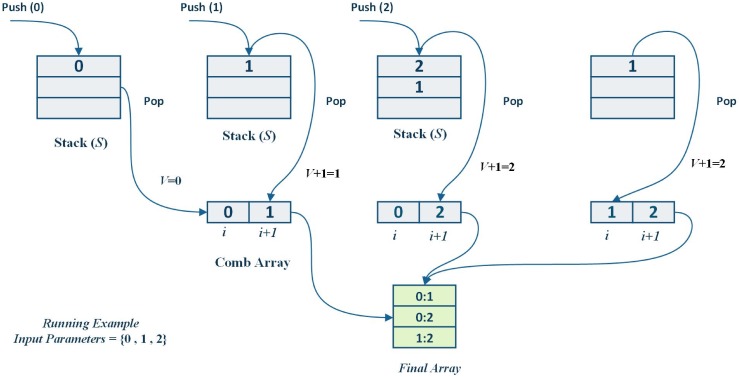
A Running Example.

Fig 5 shows a running example to illustrate how the combinations of input parameters were generated using three input parameters [0, 1, and 2]. With the first parameter pushed into the stack at start, the algorithm iterates and the stack popped its last value to the *i* + 1 index of the Comb array. In the next iteration, the stack was pushed by *v* + 1 value. The algorithm stops when the stack became empty. The final array then contains all the interaction of input parameters which are, [(0:1), (0:2), (1:2)].

As can be seen in Fig 5, the algorithm kept the previous value of *v* for the next iteration unless it became greater than the t value. For example, *v* = 0 in the first iteration and in the next iteration, it became *v* + 1, which equals to 1. Then it was incremented and pushed into the stack again.

### Test Set Generation Procedure

The generation of an optimum combinatorial test set has emerged as an active research topic in the last decade. Different strategies have been developed to address this issue especially in the software testing domain. It is also targeted as a mathematical problem to generate an optimal CA. Much recent efforts have focused on the adoption of meta-heuristic algorithms as the basis for these strategies to optimize the final set. In line with the upcoming field called Search based Software Engineering (SBSE) [49], many newly developed meta-heuristic based combinatorial strategies (e.g. based on Genetic Algorithm (GA), Ant Colony Optimization Algorithm (ACO) [50], Particle Swarm Optimization (PSO) [25], Simulated Annealing [1], and Cuckoo Search (CS) [30]).

Choosing one of the aforementioned strategies depends on different factors such as the application under test and other relevant context. When it comes to comparison among the generation strategies, the comparison is usually based on the “best solution”, which is the smallest size of the final set. In addition, in some applications, adding or extracting one test to/from the final set makes a big difference. However, in some other applications, there is a need for “good enough solution” to get an optimum set to apply for test.

Most recently, we have implemented an efficient strategy to generate CA using PSO implementation that hybridized with Fuzzy logic [19]. The strategy showed its efficiency in generating optimum CAs as compared to other strategies in many cases. In this research, we have chosen the same strategy to generate the combinatorial test set. This strategy is chosen due to three main reasons. Firstly, the strategy produces optimum results in many cases. Secondly, for the application under test in this research, we seek a “good enough solution”, which fits our developed strategy. Hence there is no need to develop a new strategy for generation since this produce optimum results. Finally, fuzzy logic takes care of parameter tuning of PSO algorithm which eases out its implementation.

**Fig 6:**
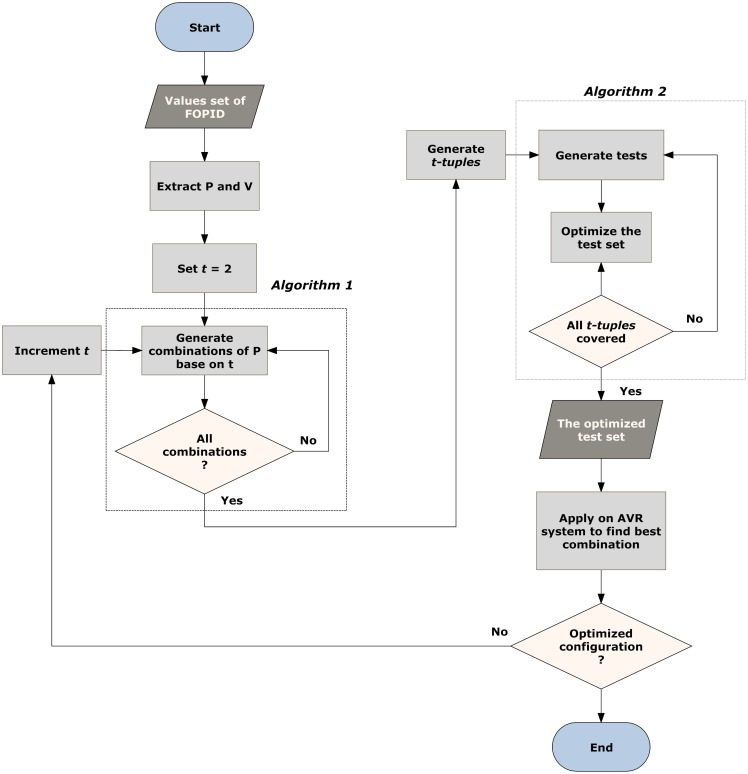
Application Steps Flow.

The overall strategy used in this research is shown in Fig 6. It takes the values set of FOPID, then manipulates them to know the number of parameters and the values belonging to each of them, denoted by P and V in Fig 6.

Initially, the combination interaction is set to the minimum strength (*t* = 2). Based on this strength, the combination of P is generated (as illustrated in Algorithm 1). Then the values are settled to each corresponding parameter to form *t* − *tuples*. Here, the process of test set generation starts with the help of the optimization algorithm to cover all the *t* − *tuples* with an optimum test set as illustrated in Algorithm 2. This optimum set will be the parameter setting for the FOPID controller. The FOPID setting will be changed based on these configurations to be applied to the AVR system. The AVR system tries these values to get the best combination of them. In case if the AVR system could not get the optimum setting of the FOPID when *t* = 2, the combination strength is increased and a new set is generated. This process is continued until the optimum setting of the FOPID is reached.

As can be seen from Algorithm 2, when *t* − *tuples* are produced, the algorithm generates a random search space based on the value range for each provided parameter. Based on our earlier implemented algorithm [19], the test sets are optimized using PSO. As shown in Algorithm 2, with this algorithm, a random search space is generated first. This set contains the possible setting of the FOPID. Each row in the search space represents a setting. To find the best candidate set, each row of this random search space undergoes through an extensive evaluation. The evaluation is based on the coverage of *t* − *tuples*. A best candidate *lBest* is the setting that can cover maximum number of *t* − *tuples*. The algorithm iterates to update the search space. The Search space is updated by the PSO update equations to approach the best settings. Instead of providing a specific number of iterations, the algorithm continues to iterate until it could not find any better solution. If after several iterations no better *lBest* can be found, then this *lBest* becomes the global best solution, *gBest*. The algorithm adds this *gBest* to the final set. To avoid repeating the coverage of the same tuples in in the *t* − *tuples* set, the tuples are removed form the set. The algorithm continues in this process until all *t* − *tuples* are covered.

**Algorithm 2**: Test Set Optimization Algorithm

 **Input**: *t* − *tuples* set, *P* and *v*

 **OutPut**: A test set

1 Store *t* − *tuple* set in a sorted hash table *H*_*t*_

2 Initialise *m* × *P* random population *S*_*P*_ where for *m* row *X*_*i*_, where *i* = 1, 2, …, *m*

3 *Iter* ← 1

4 **while** *H*_*t*_ ≠ *empty* **do**

5  **while** *Iter* < *Max. Iter* **do**

6   **foreach** *X*_*i*_ *in* *S*_*P*_ **do**

7    check coverage of *t* − *tuples*

8    return best *X*_*i*_

9   **end**

10   best *lBest* ← *X*_*i*_

11   update *S*_*P*_

12   evaluate *X*_*i*_(*t* + 1)

13   **if** *best coverage achieved by* *lBest*(*t* + 1) **then**

14    *lBest* ← *lBest*(*t* + 1)

15   **end**

16  **end**

17  *gBest* ← *lBest*(*t* + 1)

18  Add *gBest* to the test set

19  Remove all the related tuples from *H*_*t*_

20 **end**

## Results

In this section, the proposed tuning method of the FOPID controller is tested on the AVR system model defined by Eq (5). The lower and upper bounds of each FOPID controller parameter is defined within the ranges: 0 ≤ *K*_*p*_ ≤ 3, 0 ≤ *K*_*i*_ ≤ 1, 0 ≤ *K*_*d*_ ≤ 1, 0 ≤ λ ≤ 2 and 0 ≤ *μ* ≤ 1. The parameters of the Oustaloup approximation are chosen to be *ω*_*l*_ = 0.001*ω*_*c*_, *ω*_*h*_ = 1000*ω*_*c*_ where *ω*_*c*_ is the gain cross frequency, and N = 6. An optimal solution vector of the FOPID parameters, $k=\{K_{p}^{*},K_{i}^{*},K_{d}^{*},\lambda^{*},\mu^{*}\}$ is defined in a five dimensional real domain $R^{5}$. Then a suboptimal solution vector $\vec{k_{s}}=\left\{K_{p}^{s*},K_{i}^{s*},K_{d}^{s*},\lambda^{s*},\mu^{s*}\right\}$, is defined in a discrete five dimensional real domain $R_{d}^{5}$ such that,

$$R_{d}^{5}=\left\{\left(K_{p},K_{i},K_{d},\lambda ,\mu \right)R^{5}\right\}$$ (14)

In the discrete domain, $R_{d}^{5}$, the parameters *K*_*p*_, *K*_*i*_, *K*_*d*_, λ, and *μ* are defined within the above ranges in a discrete manner with step sizes equal to 1/10, 1/30, 1/30, 1/15, and 1/15 respectively. With these discrete ranges, each parameter will have 30 possible values, thus producing a total of (30^5^ = 24300000) possible combinations.

For determining the optimum values of the gains of the controller, a weighted sum objective function $J(\vec{K})$ is defined by

$$J\left(\vec{K}\right)=\{\begin{array}{c} w_{1}M_{p}+w_{2}t_{r}+w_{3}t_{s}+w_{4}E_{ss}\;if\vec{K}\;stable\\ \;L\;if\;\vec{K}\;unstable \end{array}$$ (15)

where $\vec{K}=\left[K_{p},K_{i},K_{d},\lambda ,\mu \right]$ and *L* is a large positive real number used to penalize the fitness value of an unstable solution vector $\vec{K}$. The performance criterion Eq (15) comprises four evaluation parameter terms; overshoot *M*_*p*_, rise time *t*_*r*_, settling time *t*_*s*_, and steady state error *E*_*s*_. The significance of each term is defined by a weighting factor *w*_*i*_. For an optimum compromised response, the weighting factors in Eq (15) are selected to be; *w*_1_ = 0.452, *w*_2_ = 0.438, *w*_3_ = 0.11, and *w*_4_ = 100 [51].

**Fig 7:**
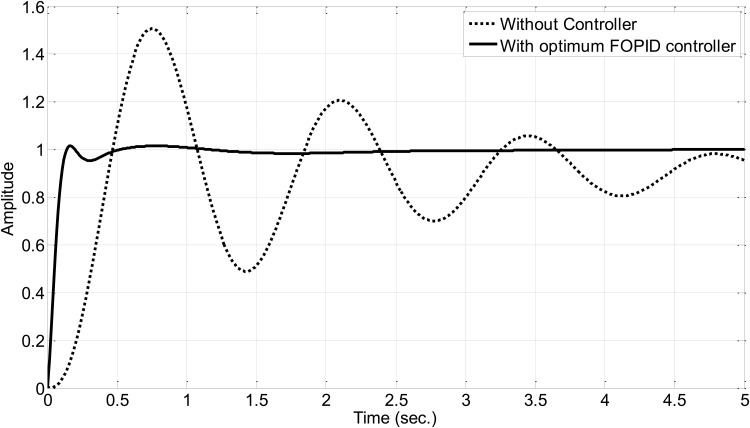
AVR system block diagram.

In an exhaustive experiment, the objective value of each possible combination within the discrete domain is calculated (calculation of 30^5^ objective values). The exhaustive experiment is conducted in a laboratory of 30 computers. Each computer is responsible to calculate a subset of 30^4^ = 810000 objective values. The subset parameter combinations are defined by one of the 30 possible values of the parameter *K*_*p*_. Among all the calculated objective values, it has been found that the optimum controller parameters vector is,

$${\vec{K}}^{*}=\left[K_{p}^{*}=2.9,K_{i}^{*}=0.73,K_{d}^{*}=0.43,\lambda^{*}=1.2,\mu^{*}=1.4\right]$$ (16)

achieving a minimum objective value $J({\vec{K}}^{*})=0.09306$. The step response of the AVR system controlled by the FOPID controller with optimum parameters is shown in Fig 7.

From the unit step response shown in Fig 7, it can be observed that *t*_*r*_ = 0.0892, *t*_*s*_ = 0.4227, *t*_*p*_ = 0.7800, and *M*_*p*_ = 1.47%. The optimal FOPID parameters are optimized to achieve minimum fitness value according to the desired response specifications.

In conventional FOPID optimization methods, such as ABC, MOL or GA, the optimization search is performed within an open space of parameters set. For each parameter, a real range of values is defined such as the ranges selected in this section. The open space search delays the optimization process, consumes its effort and may lead to local minimum problems. Therefore, combinatorial interaction design reduces the space of search and thus achieves an effective optimization process.

The table of all FOPID parameters combinations is supplied to the process of combinatorial set construction, explained in Fig 6, to produce 3 constructed sets of FOPID combinations corresponding to 2, 3, and 4-way testing. The constructed sets of the 2, 3, and 4-way sets consist of 1241, 42215, and 896528 FOPID parameters combinations and form only 0.005%, 0.174%, and 3.689% of the total number of possible combinations respectively.

**Fig 8:**
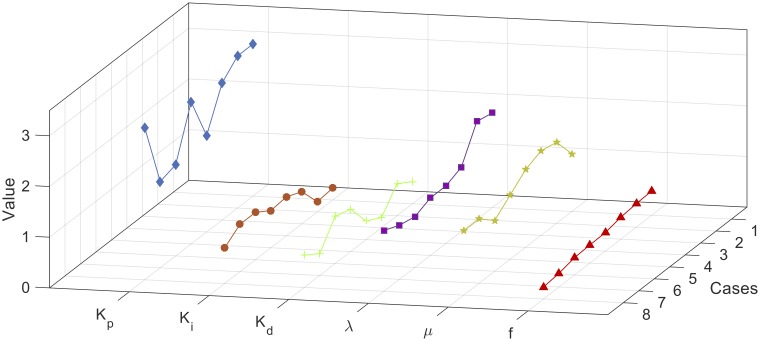
Filtered parameters combination of the constructed 2-way testing set.

**Fig 9:**
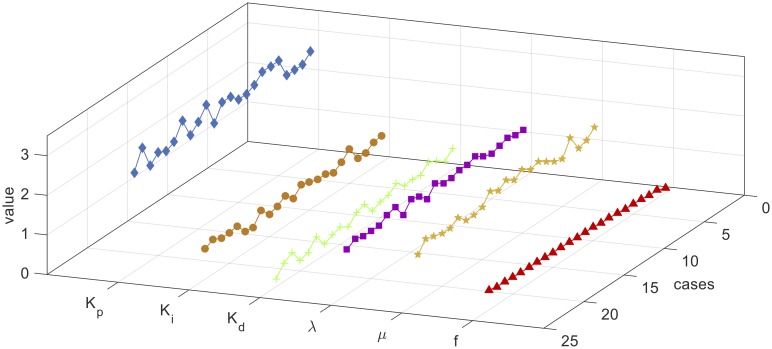
Filtered parameters combination of the constructed 3-way testing set.

**Fig 10:**
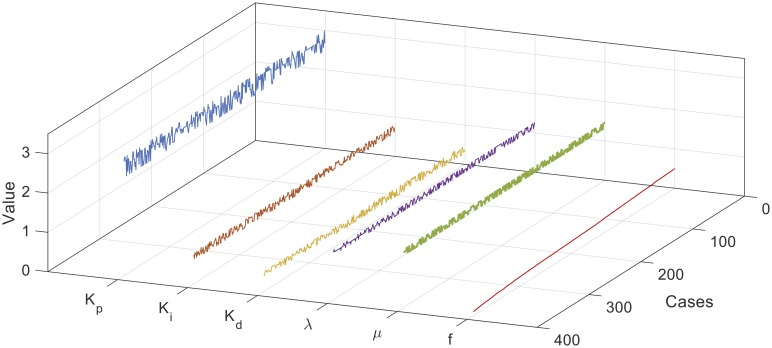
Filtered parameters combination of the constructed 4-way testing set.

The parameters combinations of each constructed set are used in the control system to calculate the corresponding fitness value using Eq (15). Thereafter, the combinations parameters are sorted by the fitness value ascendingly and filtered to exclude the combinations with fitness values greater than 0.5. Figs 8, 9 and 10 show the filtered parameters combination of the constructed 2, 3, and 4-way testing sets along with their corresponding fitness values respectively.

**Fig 11:**
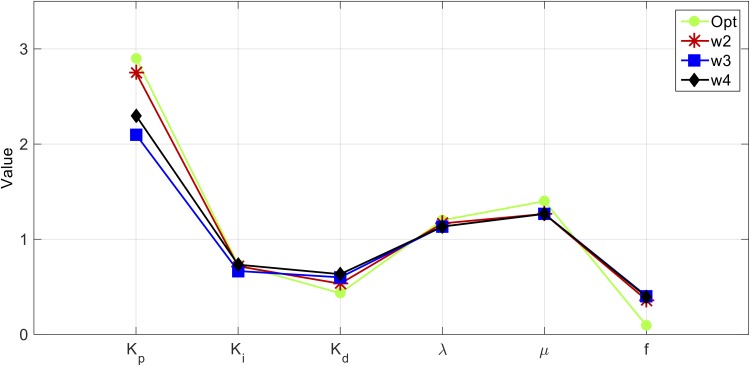
Comparison between the three cases.

From Figs 8, 9 and 10, it can be seen that the best combination parameters of the FOPID lies within the same ranges of each parameter in the three cases. In Fig 8 the parameters $K_{p}^{*},K_{i}^{*},K_{d}^{*},\lambda^{*}$ and μ* range around the median values 2.75, 0.7167, 0.533, 1.167, and 1.267, and in Fig 9 range around the median values 2.1, 0.67, 0.6, 1.13, and 1.267 respectively. For the 4-way case shown in Fig 10, the parameters range around the median values 2.3, 0.73, 0.63, 1.13, and 1.267 respectively. Fig 11 shows the median values of $K_{p}^{*},K_{i}^{*},K_{d}^{*},\lambda^{*}$ and μ* for the three cases.

## Discussion

As shown in Fig 11, the medians of the cases are comparable to the optimum values. Compared to the other cases, the medians of the 2-way case are the most proximate to the optimum. However, from all the combination parameters obtained in the 4-way case, one of the best parameter combinations equals the global optimal solution vector. Therefore, it is important to mention that the proposed method succeeds in reducing the searching domain within small ranges and assures that the global optimal solution lies within these ranges. Table 1 lists the optimum parameters combination set of the three test cases.

**Table 1:** Optimum parameters combination of the test cases.

Test Case	$K_{p}^{*}$	*K*_i_*	*K*_d_*	λ*	μ*	*f*
2-way	3	0.7	0.633333	1.2	1.266667	0.233707
3-way	2.6	0.666667	0.533333	1.2	1.466667	0.122508
4-way	**2.9**	**0.733333**	**0.43333**	**1.2**	**1.4**	**0.093057**

In the chosen discrete domain, $R_{d}^{5}$, the parameters *K*_*p*_, *K*_*i*_, *K*_*d*_, λ and *μ* are defined within certain ranges in a discrete manner with certain step sizes. Among all the possible parameter combinations, only one optimal set is found as shown in Fig 11. However, within the actual continuous domain, there might be a better optimal set which has not been included within the discrete domain. This actual optimal set has been excluded due to the step sizes used to generate the discrete domain. Thus, the proposed searching method can be improved to refine the optimal results in re-configuring the searching ranges near and around the discrete optimal result.

It is apparent from Table 1 that the 4-way case, indicated with bold font, has succeeded in finding the global optimum solution compared to the other cases. In summary, these results show that the higher the interaction strength of combinations, the more likely to find the optimum parameters combination. However, choosing higher interaction strength impose considerable searching efforts. In other control system applications in which the control parameters are too many, it is necessary to pick out the minimum adequate interaction strength. In future investigations, it might be possible to discover a relation between the number of parameters (the size of the design variable) and the minimum interaction strength. This is an important issue for future research.

## Conclusion

In this paper, we have presented our new approach to tune and find the optimal design of FOPID controller. In contrast to state of the art approaches, our approach uses the combinatorial test design method to find the optimal design. The new approach examines how the system behave under different circumstances and scenarios by combining the variables of FOPID. In addition, it helps to find the optimal design in a faster and more accurate way. To generate the combinatorial set for the tuning process, different new and effective algorithms have been implemented. A new mechanism is used to generate the combination. Problem dependent optimization is used to generate the optimal set for combinations to be tested over the system. The effectiveness of our approach is examined through a case study. This approach represents the first study to apply t-way combinatorial test design method on control engineering. Our approach marks new direction of research in using combinatorial test design method for control engineering.

Concerning future work, our approach marks new direction of research in using combinatorial test design method for control engineering. We are planning also to apply this approach to other industrial applications to investigate its effectiveness. For example, the approach could be useful for material design when different combinations must be tested to get best quality material. Another direction of research using this approach is to test security of systems by taking combinations of input factors.
